# Phylogenetic Analyses of Rotavirus A from Cattle in Uruguay Reveal the Circulation of Common and Uncommon Genotypes and Suggest Interspecies Transmission

**DOI:** 10.3390/pathogens9070570

**Published:** 2020-07-14

**Authors:** Matías Castells, Rubén Darío Caffarena, María Laura Casaux, Carlos Schild, Samuel Miño, Felipe Castells, Daniel Castells, Matías Victoria, Franklin Riet-Correa, Federico Giannitti, Viviana Parreño, Rodney Colina

**Affiliations:** 1 Laboratorio de Virología Molecular, CENUR Litoral Norte, Centro Universitario de Salto, Universidad de la República, Rivera 1350, Salto 50000, Uruguay; matvicmon@yahoo.com; 2 Instituto Nacional de Investigación Agropecuaria (INIA), Plataforma de Investigación en Salud Animal, Estación Experimental la Estanzuela, Ruta 50 km 11, Colonia 70000, Uruguay; rdcaffarena@gmail.com (R.D.C.); mlcasaux@gmail.com (M.L.C.); schild.co@gmail.com (C.S.); frcorrea@inia.org.uy (F.R.-C.); fgiannitti@inia.org.uy (F.G.); 3 Facultad de Veterinaria, Universidad de la República, Alberto Lasplaces 1620, Montevideo 11600, Uruguay; 4 Sección de Virus Gastroentéricos, Instituto de Virología, CICVyA, INTA Castelar, Buenos Aires 1686, Argentina; mino.samuel@inta.gob.ar (S.M.); vivipar3015@gmail.com (V.P.); 5 Doctor en Veterinaria en Ejercicio Libre, Asociado al Laboratorio de Virología Molecular, CENUR Litoral Norte, Centro Universitario de Salto, Universidad de la República, Rivera 1350, Salto 50000, Uruguay; felicastells@gmail.com; 6 Centro de Investigación y Experimentación Dr. Alejandro Gallinal, Secretariado Uruguayo de la Lana, Ruta 7 km 140, Cerro Colorado, Florida 94000, Uruguay; castells@adinet.com.uy

**Keywords:** rotavirus, bovine, genotypes, interspecies transmission, diarrhea

## Abstract

Uruguay is one of the main exporters of beef and dairy products, and cattle production is one of the main economic sectors in this country. Rotavirus A (RVA) is the main pathogen associated with neonatal calf diarrhea (NCD), a syndrome that leads to significant economic losses to the livestock industry. The aims of this study are to determine the frequency of RVA infections, and to analyze the genetic diversity of RVA strains in calves in Uruguay. A total of 833 samples from dairy and beef calves were analyzed through RT-qPCR and sequencing. RVA was detected in 57.0% of the samples. The frequency of detection was significantly higher in dairy (59.5%) than beef (28.4%) calves (*p* < 0.001), while it did not differ significantly among calves born in herds that were vaccinated (64.0%) or not vaccinated (66.7%) against NCD. The frequency of RVA detection and the viral load were significantly higher in samples from diarrheic (72.1%, 7.99 log_10_ genome copies/mL of feces) than non-diarrheic (59.9%, 7.35 log_10_ genome copies/mL of feces) calves (*p* < 0.005 and *p* = 0.007, respectively). The observed G-types (VP7) were G6 (77.6%), G10 (20.7%), and G24 (1.7%), while the P-types were P[5] (28.4%), P[11] (70.7%), and P[33] (0.9%). The G-type and P-type combinations were G6P[11] (40.4%), G6P[5] (38.6%), G10P[11] (19.3%), and the uncommon genotype G24P[33] (1.8%). VP6 and NSP1-5 genotyping were performed to better characterize some strains. The phylogenetic analyses suggested interspecies transmission, including transmission between animals and humans.

## 1. Introduction

Neonatal calf diarrhea (NCD) is a syndrome of worldwide distribution and the major cause of mortality of dairy calves before weaning [1]. NCD has a negative impact on animal welfare and leads to significant economic losses to the livestock industry [2,3,4,5].

Rotavirus A (RVA) is the main pathogen associated with NCD [6,7]. RVA (species *Rotavirus A*; genus *Rotavirus*; subfamily *Sedoreovirinae*; family *Reoviridae*) is a nonenveloped virus with a triple-layered capsid and a genome composed of 11 segments of double-stranded RNA [8]. RVA is widespread in dairy farms in Uruguay, and viable viral particles have been detected in sources of drinking water used for calves [9], suggesting water contamination and waterborne transmission.

Rotaviruses are classified by a binary system of G and P types for VP7 and VP4, respectively, determined by sequence analyses. In 2008, a complete genome classification system, named genotype constellation, assigning a specific genotype to each of the 11 genome segments was developed [10]. The VP7-VP4-VP6-VP1-VP2-VP3-NSP1-NSP2-NSP3-NSP4-NSP5/6 genes of rotavirus strains are classified using the abbreviations Gx-P[x]-Ix-Rx-Cx-Mx-Ax-Nx-Tx-Ex-Hx (where x is the genotype number), respectively.

Recently, since the inclusion of gene segments other than VP7 and VP4 in molecular analyses, gene reassortment has been described as a common event in RVA, sometimes between virus strains originated from different hosts, suggesting interspecies transmission [10,11,12,13].

Surveys describing the epidemiology of RVA in cattle in South America are mainly restricted to Brazil and Argentina; no published data about RVA epidemiology in Uruguayan calves are available. However, other viruses such as bovine coronavirus and bovine astrovirus have been detected in Uruguay [14,15].

Uruguay is one of the main exporters of beef [16] and dairy products [17]. Furthermore, cattle production is one of the main economic sectors in this country, with almost 12 million head of cattle accounting for 33% of the total exports [18]. The aims of this study are to determine the frequency of RVA infections and to analyze the genetic diversity of the RVA strains detected in Uruguayan calves.

## 2. Results

### 2.1. Detection Frequency of RVA in Uruguayan Calves

**Figure 1:**
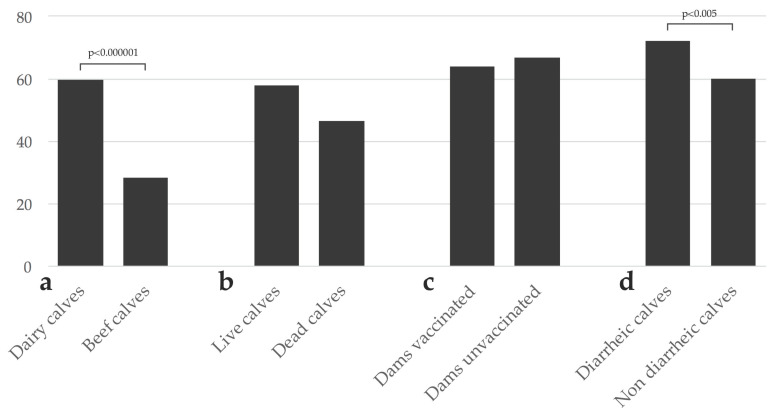
Frequency of Rotavirus A (RVA) detection in calves. (**a**) Frequency of RVA detection in dairy vs. beef calves; (**b**) frequency of RVA detection in live vs. deceased calves; (**c**) frequency of RVA detection in calves from vaccinated ^a^ vs. unvaccinated dairy herds; (**d**) frequency of RVA detection in diarrheic vs. non diarrheic dairy calves. Comparisons with statistically significant differences are indicated. ^a^ Most of the vaccines against neonatal calf diarrhea available in Uruguay include two RVA strains.

Rotavirus A was detected in 57.0% (475/833) of the analyzed samples. The frequency of detection was significantly higher in dairy (59.5%, 456/766) than beef (28.4%, 19/67) calves (OR: 3.72, 95% CI: 2.14–6.44; *p* < 0.000001; Figure 1a). The frequency of RVA detection in live calves was higher (58.0%, 444/766) than in deceased calves (46.3%, 31/67), although this difference was not statistically significant (*p* = 0.06; Figure 1b). The frequency of detection in dairy calves born in herds that vaccinated (64.0%, 144/225) or did not vaccinate dams (66.7%, 164/246) against NCD did not differ significantly (*p* = 0.5; Figure 1c). The frequency of RVA detection was significantly higher in samples from diarrheic (72.1%, 173/240) than non-diarrheic (59.9%, 163/272) dairy calves (OR: 1.73, 95% CI: 1.19–2.50; *p* < 0.005; Figure 1d). No seasonal distribution was observed in RVA detection (data not shown).

Rotavirus A was detected in 58.8% (87/148), 70.6% (142/201), 68.2% (75/110), and 52.9% (18/34) of dairy calves in the first, second, third, and fourth weeks of life, respectively (Table 1). Statistically significant differences were observed between the second and the first weeks of age (OR: 1.69, 95% CI: 1.08–2.64; *p* = 0.02), and between the second and the fourth weeks of age (OR: 2.14, 95% CI: 1.02–4.48; *p* = 0.04). The mean age in days of RVA-positive dairy calves was significantly lower in diarrheic than nondiarrheic calves (*p* = 0.02; Table 1).

The RVA viral load was significantly higher in diarrheic than nondiarrheic dairy calves (*p* = 0.007; Table 1), ranging between 1.14 × 10^4^ and 7.36 × 10^12^ genome copies/milliliter (gc/mL) of feces. In all four age groups, the frequency of RVA detection was higher in diarrheic than nondiarrheic dairy calves: 69.0% (40/58) vs. 52.2% (47/90) in the first week, 72.1% (98/136) vs. 67.7% (44/65) in the second week, 68.8% (22/32) vs. 67.9% (53/78) in the third week, and 85.7% (6/7) vs. 44.4% (12/27) in the fourth week of age. A statistically significant difference was observed only within the first week (OR: 2.03, 95% CI: 1.01–4.07; *p* = 0.04).

### 2.2. VP7 and VP4 Genotyping

We obtained 58 and 116 sequences for VP7 and VP4, respectively. The detected G-types (VP7) were G6 (77.6%, 45/58), G10 (20.7%, 12/58), and G24 (1.7%, 1/58), while the P-types (VP4) were P[5] (28.4%, 33/116), P[11] (70.7%, 82/116), and P[33] (0.9%, 1/116). The following G- and P-type combinations were obtained for 57 strains: G6P[11] (40.4%, 23/57), G6P[5] (38.6%, 22/57), G10P[11] (19.3%, 11/57), and G24P[33] (1.8%, 1/57). Furthermore, 60 strains had undetermined G- or P-type: GXP[11] (80.0%, 48/60), GXP[5] (18.3%, 11/60), and G10P[X] (1.7%, 1/60).

### 2.3. VP6 and NSP1-5 Genotyping

Ten samples, including representative VP7 and VP4 genotype combinations observed, were selected for VP6 and NSP1-5 gene characterization: 2 G6P[5], 2 G6P[11], 2 G10P[11], 2 GXP[11], 1 G10P[X], and 1 G24P[33] (Table 2). All the strains were I2 (VP6), N2 (NSP2), and E12 (NSP4). Nine were H3 and one could not be determined HX (NSP5). Five strains were A3, four were A13, and one could not be determined AX (NSP1). Eight strains were T6, one was T9, and one could not be determined TX (NSP3). 

### 2.4. Phylogenetic Analyses

**Figure 2:**
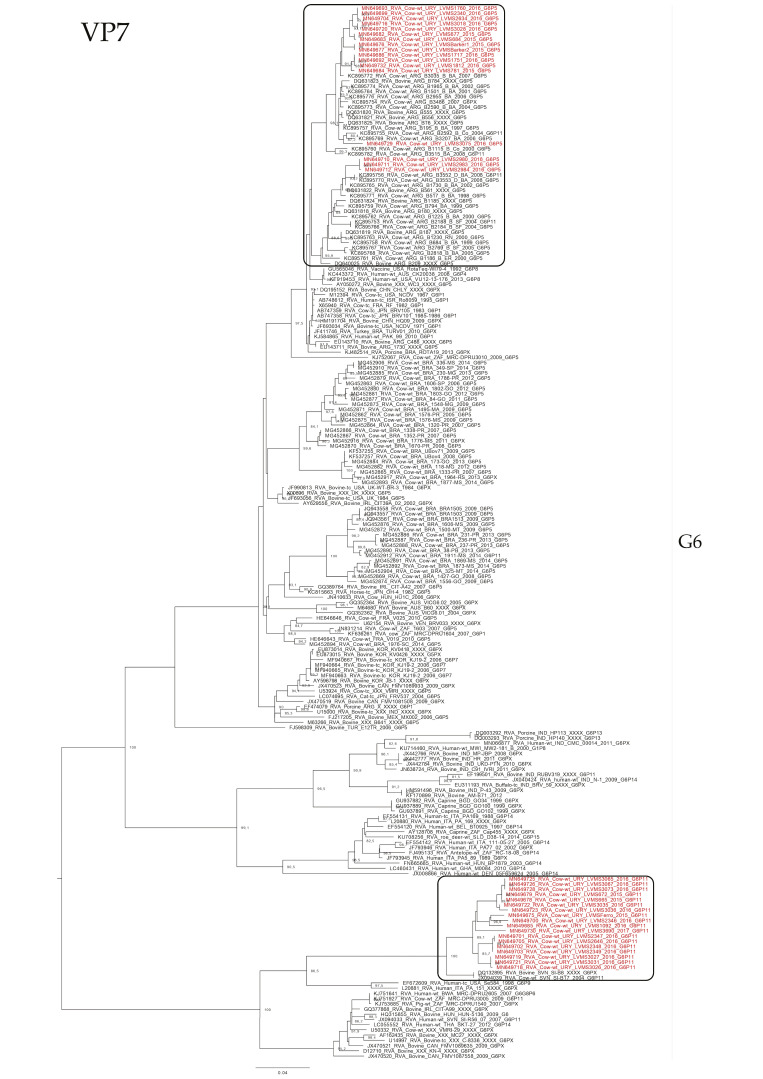
Maximum likelihood tree of the G6 genotype of the VP7 gene. The best nucleotide substitution model (TIM2 + I + G) and the maximum likelihood tree were obtained with W-IQ-TREE. Uruguayan strains are shown in red. Shimodaira–Hasegawa-approximate likelihood-ratio test (SH-aLRT) values ≥ 80 are shown.

**Figure 3:**
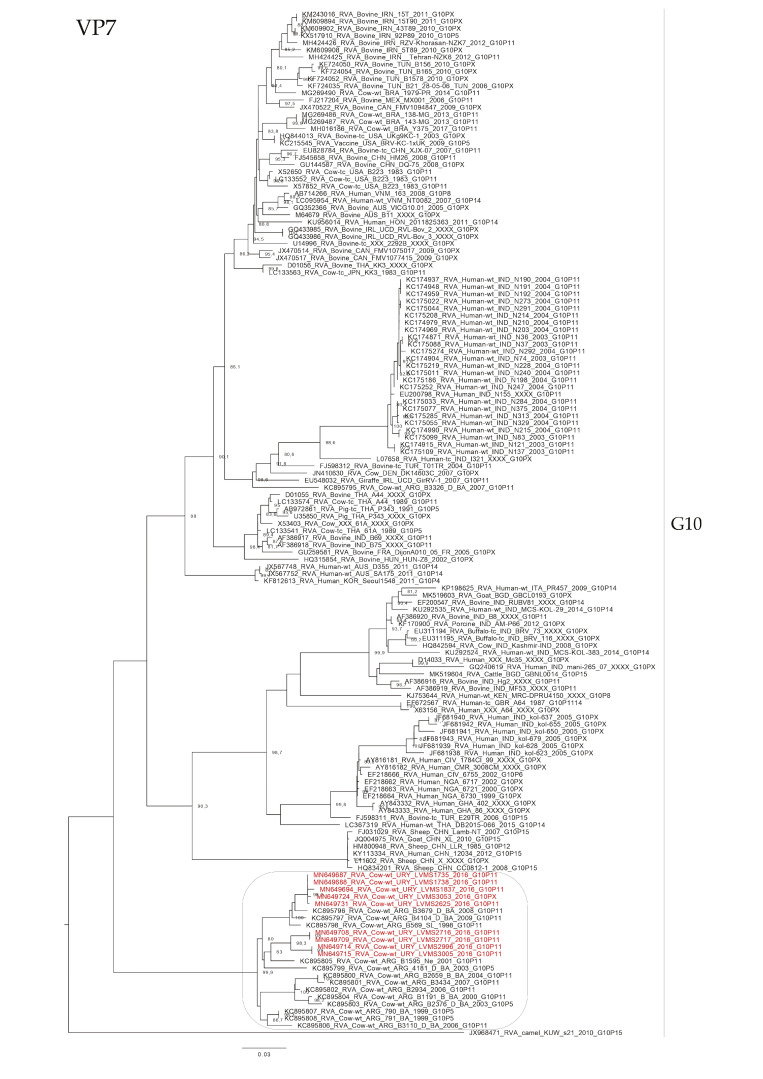
Maximum likelihood tree of the G10 genotype of the VP7 gene. The best nucleotide substitution model (TPM3 + G) and the maximum likelihood tree were obtained with W-IQ-TREE. Uruguayan strains are shown in red. SH-aLRT values ≥ 80 are shown.

The phylogenetic analyses showed an intricate genetic scenario. The analyses of the VP7 gene showed that G6 and G10 Uruguayan strains clustered in two and one different lineages, respectively, with sequences obtained from cattle. Specifically, the G6P[5] Uruguayan strains clustered in one lineage (split into two sublineages) with Argentinian strains, and the G6P[11] Uruguayan strains clustered separately in a lineage with Slovenian strains (Figure 2). The G10 Uruguayan strains clustered in a lineage (split into two sublineages) with Argentinian strains (Figure 3). Brazilian G6 and G10 strains clustered separately with Uruguayan and Argentinian G6 and G10 strains.

**Figure 4:**
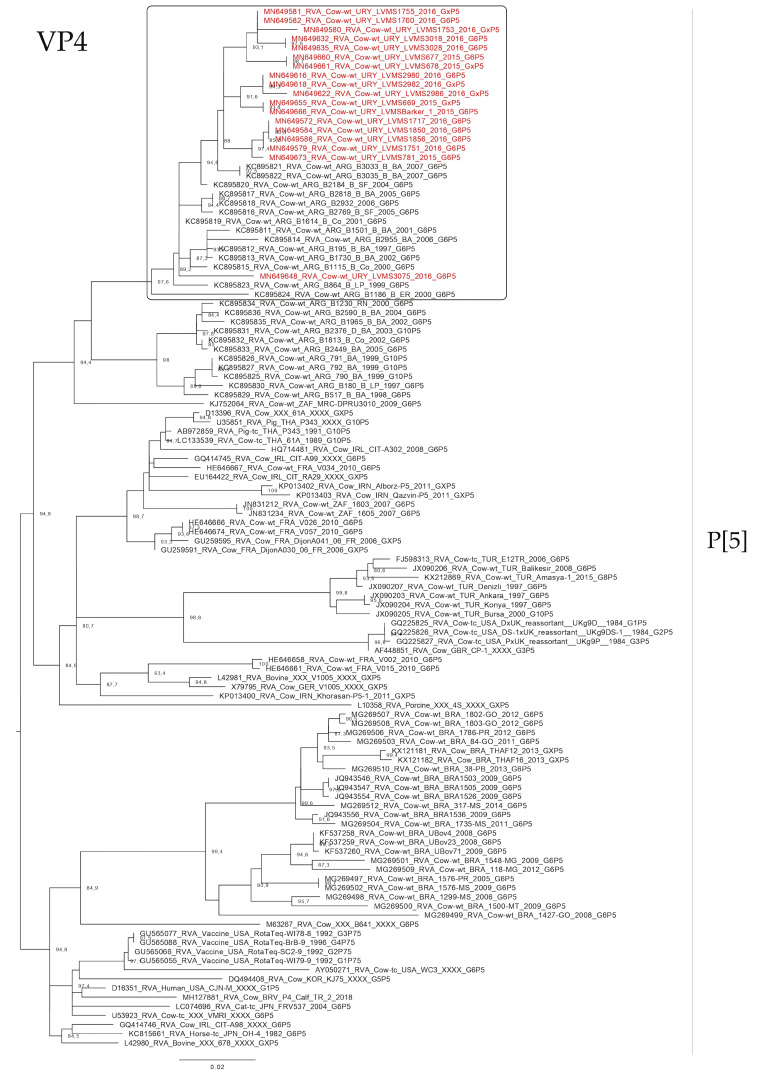
Maximum likelihood tree of the P[5] genotype of the VP4 gene. The best nucleotide substitution model (TIM + G) and the maximum likelihood tree were obtained with W-IQ-TREE. Uruguayan strains are shown in red. SH-aLRT values ≥ 80 are shown.

**Figure 5:**
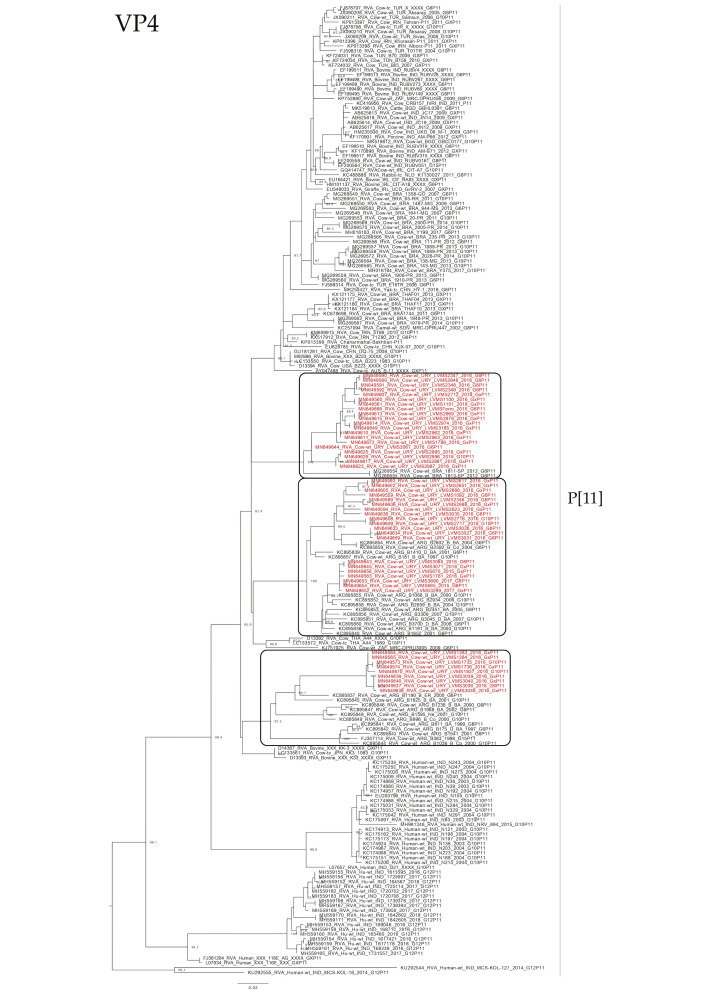
Maximum likelihood tree of the P[11] genotype of the VP4 gene. The best nucleotide substitution model (TPM3u + G) and the maximum likelihood tree were obtained with W-IQ-TREE. Uruguayan strains are shown in red. SH-aLRT values ≥ 80 are shown.

The phylogenetic analyses of the VP4 gene showed that P[5] Uruguayan strains clustered in a lineage with Argentinian G6P[5] strains obtained from cattle, and Brazilian P[5] strains clustered separate (Figure 4). The P[11] Uruguayan strains clustered in three lineages with sequences obtained from cattle, two of the lineages were comprised of G6 and G10 Argentinian strains (and one of these lineages is split into two sublineages), and the other lineage comprised of G6P[11] Brazilian strains, although P[11] Uruguayan strains were distinct to the majority of the Brazilian P[11] strains (Figure 5).

In the phylogenetic tree of the NSP1 gene, we observed that Uruguayan strains clustered in three different genetic lineages of the genotype A3: one jointly with human strains from Paraguay and Brazil, another with Italian and Belgian human strains, and another with a goat strain from Argentina and, in one genetic lineage of the genotype A13, with an Argentinian strain from a cow (Figure S1).

The phylogenetic analysis of the NSP2 gene showed that the Uruguayan strains were clustered in two separate lineages: one with Argentinian strains from cow and goat, and the other with strains from guanaco and vicuña from Argentina and strains from humans from Australia (Figure S2). 

On the other hand, the phylogenetic analysis of the NSP3 gene showed that the T6 Uruguayan strains were clustered in three sublineages within one lineage: one together with strains distributed worldwide (including vaccine strains), one with Argentinian (vicuña and guanaco), Japanese (cow), Slovenian (human), and Paraguayan (human) strains, and the third with a goat strain from Argentina and a human strain from Belgium. The T9 strain clustered with the other four T9 strains detected so far (from Japan and the USA; Figure S3). 

For the NSP4 gene, we observed that besides the Uruguayan strains obtained in our study, only sequences from South America were available. The phylogenetic analysis showed that Uruguayan strains clustered in four different lineages together with strains from several host species (cows, guanacos, horses, goats, and humans), all from this subcontinent (Figure S4).

The phylogenetic analysis of the NSP5 gene showed that the Uruguayan strains were clustered in three sublineages within one lineage: one together with strains distributed worldwide in several host species), other with an Argentinian strain from a cow and a Paraguayan strain obtained from a human, and another with a strain from a guanaco from Argentina, a strain from a yak from China, and a strain from a human from Hungary (Figure S5).

Lastly, the phylogenetic analysis of the VP6 gene showed that the Uruguayan strains were clustered in three lineages: one conformed only with Uruguayan strains, another lineage with an Argentinian strain from a cow, and another lineage with South American strains from various hosts (human, llama, sheep, and goat), Japanese strains from human and cow, and a roe deer Slovenian strain (Figure S6).

## 3. Discussion

Rotavirus A was detected in feces and intestinal contents collected from dairy and beef calves with a frequency of 57%, which was higher than reports from Argentina and Brazil (17–42%) [19,20,21,22], and other geographic regions (20–49%) [7,23,24,25]. On the other hand, in Australia, the frequency of RVA detection was 80%, which is higher than the detected in our study [6]. Interestingly, most of the mentioned studies were conducted by assays different than RT-qPCR, except the one conducted in Australia. It is well documented that the RT-qPCR for RVA detection has a higher sensitivity than other assays, reducing the risk of false-negatives (i.e., ELISA, electron microscopy, PAGE, immunochromatography, and conventional PCR) [6,26,27,28], which could explain the higher frequency observed in Uruguay when compared with neighboring countries while reducing the risk of false-positive results, also given its higher specificity. Furthermore, the use of RT-qPCR, which is known to detect very few genomic copies, allows pathogen detection in clinical and subclinical calves. In addition, in many field situations, the time of onset of diarrhea is not known, so the peak of pathogen shedding may have already passed, or the infection could be just settling down by the time of sampling [29]. The limit of detection in our study (10^4^ gc/mL of feces) and the higher RVA viral load in diarrheic than nondiarrheic calves are in agreement with the stated by Torres-Medina et al. [29]. On the other hand, we also observed high viral loads in some nondiarrheic calves.

Infection with RVA has long been associated with diarrhea [29,30,31], as observed in our study, where RVA detection was more frequent in diarrheic than in nondiarrheic calves, independently of their age (up to 4 weeks). Concerning the calves’ age, we observed that the proportion of calves shedding RVA was higher in the second and third weeks of age, as observed in Brazil [19,32] and elsewhere [33]. In addition, the mean age of RVA-positive calves in our study is similar to the age reported previously [31], and we observed that diarrheic calves positive for RVA were younger than nondiarrheic calves, indicating that calves are exposed to this pathogen early after birth.

Although the sampling between beef and dairy farms was unequal, our results indicate that the circulation of RVA was higher in dairy than beef calves. This contrasts with the reported results in neighboring countries, where RVA was more frequently detected in beef than dairy calves [19,20] or in a similar frequency [21]. Our results also contrast with those observed in a study conducted in Australia [6].

A common practice used to prevent NCD is the vaccination of pregnant cows/heifers during the last stage of pregnancy to protect the calves by the transference of passive maternal antibodies through colostrum intake. Most available vaccines in the Uruguayan market include bovine rotavirus A strains (most of them include two strains, G6 and G10, as detailed by the manufacturers). In this study, we observed a similar frequency of RVA detection in calves from vaccinated and unvaccinated herds. Failure in the protection against RVA infection by the vaccine was reported in studies conducted in Argentina and Brazil [34,35,36,37]; although vaccines are not effective in preventing RVA infection, they significantly reduce morbidity, the severity of diarrhea, and mortality related to RVA [38].

In this study, we determined the RVA genotypes circulating in calves in Uruguay. Overall, the VP7 and VP4 genotypes observed in this country are the most prevalent in cattle worldwide [39], although, unexpectedly, we detected a G24P[33] strain, which thus far had only been reported from an asymptomatic cow and her calf in Japan [11]. The G24P[33] strain detected in Uruguay was obtained from a 10-day-old asymptomatic dairy calf sampled in August 2016.

Regarding the VP6 and NSP1-5 genotyping, the Uruguayan strains, including the G24P[33], showed a relatively conserved genotype constellation I2-A3/A13-N2-T6/T9-E12-H3, corresponding to VP6 and NSP1-5 genotypes, respectively. These genotypes are commonly found in cattle, with the exception of T9 [40]. The T9 genotype has been sporadically detected in two cows from Japan [11], in a child from Japan [41], and in a child from the USA [42]. This genotype has been associated with atypical VP7 and VP4 genotypes (G21P[29], G24P[33], G8P[14], and G24P[14]). In this study, we observed the T9 genotype associated with G24P[33]. Indepth analysis of the RVA/Cow-wt/URY/LVMS3024/2016/G24P[33] strain revealed almost the same genotype constellation as the RVA/Cow-wt/JPN/Dai-10/2007/G24P[33] strain from Japan, with the unusual G24, P[33], and T9 genotypes. The only difference was observed in the NSP4 gene that was E12 in the Uruguayan strain and E2 in the Japanese. It is interesting to note that all the Uruguayan strains were E12, a genotype widely detected in cattle [12], guanacos [12], horses [43,44], goats [45], and children [46,47] in South America. This reinforces the notion that the E12 genotype may be restricted to South America, as previously postulated [44].

The rare G24P[33] strain detected in our study represented a challenge. The G24, P[33], and T9 genotypes observed in this strain provides information for a possible introduction of the virus from Japan to Uruguay, or vice versa. The expansion of the Wagyu beef industry beyond Japan [48] could have influenced the dispersion of some RVA strains through live cattle exports. On the other hand, the E12 genotype in the Uruguayan G24P[33] strain and E2 genotype in the Japanese G24P[33] strain represented a probable gene reassortment, which is a more plausible scenario than the emergence of two independent strains with the same rare genotype constellation except for NSP4. Further studies should be conducted to determine the evolution and possible emergence of these rare genotypes.

In the phylogenetic analyses of all the genes, it can be observed that Uruguayan strains clustered mainly with South American strains. The only gene that did not show any South American-specific lineage was NSP3, in which the Uruguayan strains clustered mainly with Argentinian strains, but also with strains from other continents. These data, together with the identification of the E12 genotype in all the Uruguayan sequences, suggest a South American origin of RVA lineages [44]. Furthermore, the phylogenetic analyses showed an intricate pattern of diversity, with evidence of gene reassortments, interspecies transmission, local dispersion of some strains, and circulation of strains that are most prevalent in cattle worldwide.

The analyses of VP7 and VP4 showed a conserved pattern with all the Uruguayan strains clustering, with strains detected only in cattle and mainly from Argentina, indicating a probable host species and geographic linkage. Due to the shortage of G24 and P[33] sequences in the database (2 and 1, respectively), no phylogenetic analyses were performed for these genotypes. In the VP7 and VP4 phylogenetic analysis, the majority of strains characterized in this study clustered closely with strains detected in Argentinian cattle. The exceptions were one G6 lineage that clustered with European strains isolated from cattle, and one in P[11] sublineage that clustered with Brazilian strains isolated from cattle. There is a clear phylogenetic relationship between the strains detected in the cattle in Uruguay and Argentina, whereas Brazilian strains were, in general, phylogenetically distant from the Uruguayan strains. In addition, Uruguayan strains clustered together among themselves, suggesting that limited introductions of RVA into the country have occurred, but the strains were widely dispersed in the cattle. A possible explanation for the genetic similarity between the Uruguayan and Argentinian strains and their divergence to the Brazilian strains could be explained, in part, by the breed of cattle. In Uruguay and Argentina, most of the cattle breeds are *Bos taurus*, while in Brazil, there are mostly *Bos indicus* or *Bos indicus* x *Bos taurus* crosses. Although it has not been studied in cattle, different human subpopulations appeared to have different susceptibility infection and clinical disease, and this susceptibility is dependent on the rotavirus genotype, and in some cases, it also depends on different rotavirus strains of the same genotype [49].

Based on the phylogenetic analyses, we observed evidence of gene reassortment and interspecies transmission events. Regarding the former event, in addition to the previously mentioned gene reassortment of the G24P[33] strain, strong evidence was observed in the strains RVA/Cow-wt/URY/LVMS1812/2016/G6P[5] and RVA/Cow-wt/URY/LVMS3206/2016/GxP[11] because both strains clustered together in all the genes, except in VP4 (which showed different genotypes, (P[5] and P[11], respectively), indicating that a possible gene reassortment event may have occurred. Another piece of evidence was observed in the RVA/Cow-wt/URY/LVMS1788/2016/GxP[11] strain because it clustered together with other Uruguayan strains in most of the genes, except in NSP1 and NSP3 genes, which clustered alone in different genetic lineages, also suggesting a gene reassortment event. Furthermore, the strain RVA/Cow-wt/URY/LVMS1837/2016/G10P[11] clustered together with RVA/Cow-wt/URY/LVMS2625/2016/G10P[11] and RVA/Cow-wt/URY/LVMS3053/2016/G10P[x] in most of the genes, but clustered separately in distant genetic lineages in NSP2 and NSP5; this was probably due to gene reassortment. On the other hand, an interesting observation was that, in general, G6 strains tended to cluster together in most of the genes, and the same was observed for the G10 strains, with the exceptions aforementioned.

Regarding interspecies transmission, we observed that in the analyses of VP7 and VP4, all the Uruguayan strains clustered with other bovine strains, so these gene segments seem to be more host-specific than the other genes. On the other hand, and based on the phylogenetic analyses, we observed evidence suggesting interspecies transmission because the bovine strains detected in Uruguay closely clustered with strains detected in other host species. We observed that bovine Uruguayan strains A13 (NSP1 gene) clustered together with strains isolated from humans and a goat, possibly indicating events of interspecies transmission. Two lineages showed a close relationship between Uruguayan bovine strains and human strains (from South America and Europe); these human strains were reported to be Artiodactyl-like and a product of interspecies transmission [10,47,50], as well as the goat strain of a third lineage [45], which is in accordance with our results. In the NSP2-5 and VP6 genes, we observed that the Uruguayan bovine strains clustered in some lineages with strains isolated from other host species (human, goat, guanaco, vicuna, roe deer, llama, and sheep), mainly from South America, that were proposed to be originated by interspecies transmission [12,45,47,51], again in accordance with our results. Another piece of evidence supporting this event was observed in the NSP4; all the RVA strains detected in South America were E12, independent of the host species where they were isolated (horse, cow, guanaco, human, goat), suggesting interspecies transmission and fixation of this genotype in South America [44]. The interspecies transmission of RVA is widely documented [10,11,12,13], and our results support this event. In South America, it is common to raise different livestock species on the same farm in close contact with humans [45], which increases the possibility of interspecies transmission. Our results support that interspecies transmission is a common event in South America, including the possibility of zoonotic transmission [45,51,52].

Lastly, our study had some limitations. In Uruguay, dairy farming is concentrated in the southwest region and calves are raised under intensive production systems that facilitated the collection of the samples, while beef calves are mostly bred in extensive production systems and dispersed throughout the country, which hindered the access to samples. This resulted in an overrepresentation of dairy (92%) versus beef (8%) samples in our study. Another limitation was that we had no spiked control to determine if there was inhibition of the qPCR, which may lead to false-negatives. Regarding coinfections, the methodology used has the limitation that sequences obtained from a single animal would have only represented the predominant strain and/or sequences with multiple traces that were not included in the study. It is important to mention that, from our analyses, we could not determine the route nor the time in which the gene reassortment and the interspecies transmission events took place.

## 4. Materials and Methods 

### 4.1. Samples

**Figure 6:**
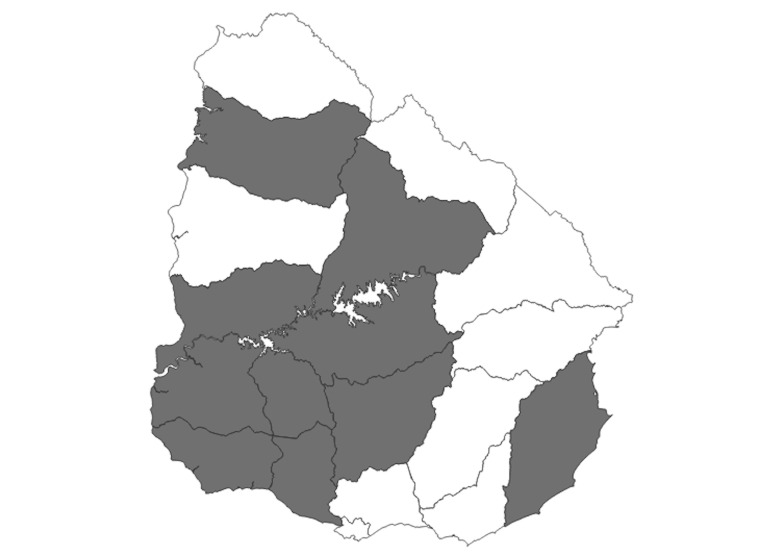
Map of Uruguay, the regions from which samples were collected shown in grey.

Fecal samples of 766 live calves and intestinal contents from 67 naturally-deceased calves were collected from 833 different calves from dairy and beef herds in Uruguay between 2015 and 2018. Sampled herds were distributed in 10 of the 19 regions of the country (Figure 6), and throughout the year, including samples collected in the four climate seasons. In addition, 766 samples were from dairy calves, and 67 from beef calves. We compared the frequency of the RVA infection between groups only for dairy calves. A total of 240 dairy calves had diarrhea at the time of sampling, while 272 were nondiarrheic dairy calves (this information was unavailable for 321 calves). The distribution by age in the first, second, third, and fourth weeks of life was 148, 201, 110, and 34 dairy calves, respectively (the age was unavailable for 340 calves). A total of 225 calves were from dairy herds vaccinated against NCD and 246 calves were from nonvaccinated dairy herds (herd vaccination history was unavailable for 362 calves).

### 4.2. Sample Suspension, RNA Extraction, Reverse Transcription, Detection and Quantification of RVA

Samples were diluted 1:10 (*v*:*v*) in phosphate-buffered saline solution, centrifuged at 3000× *g* for 20 min at 4 °C, and supernatants were collected and stored at −80 °C. Viral RNA was extracted using a QIAamp^®^ cador^®^ Pathogen Mini Kit (Qiagen^®^, Hilden, Germany), following the manufacturer’s instructions. Reverse transcription (RT) was carried out with RevertAid^®^ Reverse Transcriptase (Thermo Fisher Scientific^®^, Waltham, MA, USA) and random hexamers primers (Qiagen^®^), following the manufacturer´s instructions. All RNAs and cDNAs were stored at −80 °C until further viral analyses. Screening and quantification of the samples for RVA identification were carried out through a quantitative polymerase chain reaction (qPCR) targeted to the NSP3 gene, as described elsewhere [9]. Briefly, 12.5 µL of SensiFAST™ Probe No-ROX Kit (Bioline^®^, London, UK), 5.0 µL of nuclease-free water, 1.0 µL of 10 µM forward primer, 1.0 µL of 10 µM reverse primer, 0.5 µL of 10 µM probe, and 5 µL of cDNA were mixed in 0.2-mL PCR tubes. All samples were analyzed in duplicate. In order to validate the complete process, an RVA-positive (G6P[5] strain) and an RVA-negative fecal sample were used as positive and negative controls, respectively.

### 4.3. Rotavirus A Genotyping

Quantitative-PCR positive samples were subsequently subjected to amplification of VP7 and VP4 (VP8*). Briefly, 12.5 µL of MangoMix™ (Bioline^®^), 5 µL of cDNA, 5.5 µL of nuclease-free water, 1 µL of dimethyl sulfoxide, 0.5 µL of 20 µM forward primer and 0.5 µL of 20 µM reverse primer were mixed in 0.2-mL PCR tubes. Forward and reverse primers for VP7 and VP4 (VP8*) amplification are described elsewhere [20,53]. In addition, 10 samples, including representative VP7 and VP4 genotype combinations observed in this study, were selected for VP6 and NSP1-5 gene characterization. Primers and cycling conditions were used, as described elsewhere [10], and PCR reagents were used, as described above. Genotyping was performed using the web-based genotyping tool RotaC v2.0 [54].

### 4.4. PCR Product Purification, Sequencing, and GenBank Accession Numbers

PCR products were visualized in 1–2% agarose gels and positive samples were purified using PureLink™ Quick Gel Extraction and PCR Purification Combo Kit (Invitrogen^®^, Carlsbad, CA, USA), according to the manufacturer’s instructions. Both cDNA strands were sequenced by Macrogen Inc. (Seoul, Korea). Sequences were deposited in GenBank with accession numbers: MN649559—MN649674 (VP4), MN649675—MN649732 (VP7), MN649733—MN649742 (VP6), MN649743—MN649751 (NSP1), MN649752—MN649761 (NSP2), MN649762—MN649770 (NSP3), MN649771—MN649780 (NSP4), and MN649781—MN649789 (NSP5).

### 4.5. Phylogenetic Analysis

All the available sequences corresponding to the genotypes observed in the RVA strains detected in this study, previously determined with RotaC, were downloaded from the Virus Variation Resource (http://www.ncbi.nlm.nih.gov/genome/viruses/variation/) [55]. A dataset was created for each genotype, and multiple sequence alignments were obtained using Clustal W implemented in MEGA 7 software [56]. The final alignment of each gene comprised all the worldwide sequences that covered the length of the sequences obtained in this study. The length of the sequences and the nucleotide position, involved in the phylogenetic analysis of each gene, are detailed in Table 3. The nucleotide substitution models that best fit each dataset (Table 3) and the maximum likelihood trees were obtained using W-IQ-TREE (available at http://iqtree.cibiv.univie.ac.at) [57]. The branches support was estimated with the Shimodaira–Hasegawa-approximate likelihood-ratio test (SH-aLRT) [58]. Trees were visualized in FigTree (http://tree.bio.ed.ac.uk/software/figtree/).

### 4.6. Statistical Analyses

Data were organized and graphics were generated using Microsoft^®^ Office Excel. Categorical data were evaluated with RStudio v1.0.136 software through Pearson’s chi-squared tests. Odds ratios (OR) and 95% confidence intervals (CI) were calculated with jamovi software (available at https://www.jamovi.org/). Viral load values (genome copies/milliliter of feces) were log10 transformed. For the viral load and mean age analyses, the Shapiro–Wilk test was performed, rejecting the normality of the data, so the Mann–Whitney U test was performed with the same software. For all tests, differences were considered statistically significant if the obtained *p*-value was < 0.05.

## 5. Conclusions

Rotavirus A is widespread in cattle in Uruguay and is associated with diarrhea in calves, with a peak of viral shedding at 2–3 weeks of age, and higher viral shedding in diarrheic versus non-diarrheic calves. Even though the main genotypes observed in this country are the most prevalent worldwide, a rare strain was detected with a G24-P[33]-I2-A13-N2-T9-E12-H3 genotype constellation. The E12 genotype detected in all strains, regardless of the VP7 and VP4 genotypes, appears to be a South American geographic marker. An intricate genetic scenario was evidenced, with gene reassortment and interspecies transmission events, including transmission between animals and humans.

## Figures and Tables

**Table 1:** Frequency of RVA detection and viral load in feces of diarrheic and nondiarrheic calves.

			Calves Age			
	Mean Age ^a^	Viral Load ^b^	First Week	Second Week	Third Week	Fourth Week
Diarrheic	11.9 ^1^	7.99 ^2^	69.0 ^3^	72.1	68.8	85.7
Non-diarrheic	13.5 ^1^	7.35 ^2^	52.2 ^3^	67.7	67.9	44.4
Total	12.7	7.67	58.8 ^4^	70.6 ^4,5^	68.2	52.9 ^5^

^a^ Mean age in days of RVA-positive calves. ^b^ Mean RVA viral load expressed as log10 of RVA genome copies per milliliter of feces. Equal numbers in superscript refer to values with statistically significant differences (*p* < 0.05).

**Table 2:** Genotype constellation of 10 RVA strains from Uruguayan calves.

Strain	VP7	VP4	VP6	NSP1	NSP2	NSP3	NSP4	NSP5
RVA/Cow-wt/URY/LVMS781/2015/G6P[5]	**G6**	**P[5]**	**I2**	**AX**	**N2**	**T6**	**E12**	**H3**
RVA/Cow-wt/URY/LVMS1788/2016/GxP[11]	**GX**	**P[11]**	**I2**	**A3**	**N2**	**T6**	**E12**	**H3**
RVA/Cow-wt/URY/LVMS1812/2016/G6P[5]	**G6**	**P[5]**	**I2**	**A3**	**N2**	**T6**	**E12**	**H3**
RVA/Cow-wt/URY/LVMS1837/2016/G10P[11]	**G10**	**P[11]**	**I2**	**A13**	**N2**	**TX**	**E12**	**H3**
RVA/Cow-wt/URY/LVMS2625/2016/G10P[11]	**G10**	**P[11]**	**I2**	**A13**	**N2**	**T6**	**E12**	**H3**
RVA/Cow-wt/URY/LVMS3024/2016/G24P[33]	**G24**	**P[33]**	**I2**	**A13**	**N2**	**T9**	**E12**	**H3**
RVA/Cow-wt/URY/LVMS3027/2016/G6P[11]	**G6**	**P[11]**	**I2**	**A3**	**N2**	**T6**	**E12**	**H3**
RVA/Cow-wt/URY/LVMS3031/2016/G6P[11]	**G6**	**P[11]**	**I2**	**A3**	**N2**	**T6**	**E12**	**H3**
RVA/Cow-wt/URY/LVMS3053/2016/G10P[x]	**G10**	**P[X]**	**I2**	**A13**	**N2**	**T6**	**E12**	**HX**
RVA/Cow-wt/URY/LVMS3206/2016/GxP[11]	**GX**	**P[11]**	**I2**	**A3**	**N2**	**T6**	**E12**	**H3**

Uncommon genotypes are shadowed in grey.

**Table 3:** Information about the final alignments obtained for the phylogenetic analyses.

	NSP1	NSP2	NSP3	NSP4	NSP5	VP4 (P[5])	VP4 (P[11])	VP6	VP7 (G6)	VP7 (G10)
Sequences lenght *	1005	954	917	528	597	645	654	1143	852	837
Genomic position *	165–1169	Complete ORF	47–963	Complete ORF	Complete ORF	130–774	124–795	Complete ORF	121–972	73–909
Best nucleotide substitution model	TIM + I + G	TIM + G	TIM3 + G	HKY + G	TN + I + G	TIM + G	TPM3u + G	TIM + I + G	TIM2 + I + G	TPM3 + G

* Reference strain: WC3.

## Supplementary Materials

The following are available online at https://www.mdpi.com/2076-0817/9/7/570/s1, Figure S1: Maximum likelihood tree of the NSP1 gene. The best nucleotide substitution model (TIM + I + G) and the maximum likelihood tree were obtained with W-IQ-TREE. Uruguayan strains are shown in different colors. SH-aLRT values ≥ 80 are shown. Figure S2: Maximum likelihood tree of the NSP2 gene. The best nucleotide substitution model (TIM + G) and the maximum likelihood tree were obtained with W-IQ-TREE. Uruguayan strains are shown in different colors. SH-aLRT values ≥ 80 are shown. Figure S3: Maximum likelihood tree of the NSP3 gene. The best nucleotide substitution model (TIM3 + G) and the maximum likelihood tree were obtained with W-IQ-TREE. Uruguayan strains are shown in different colors. SH-aLRT values ≥ 80 are shown. Figure S4: Maximum likelihood tree of the NSP4 gene. The best nucleotide substitution model (HKY + G) and the maximum likelihood tree were obtained with W-IQ-TREE. Uruguayan strains are shown in different colors. SH-aLRT values ≥ 80 are shown. Figure S5: Maximum likelihood tree of the NSP5 gene. The best nucleotide substitution model (TN + I + G) and the maximum likelihood tree were obtained with W-IQ-TREE. Uruguayan strains are shown in different colors. SH-aLRT values ≥ 80 are shown. Figure S6: Maximum likelihood tree of the VP6 gene. The best nucleotide substitution model (TIM + I + G) and the maximum likelihood tree were obtained with W-IQ-TREE. Uruguayan strains are shown in different colors. SH-aLRT values ≥ 80 are shown.
