# Supplementary figures and images for: Phylogenetic Analyses of Rotavirus A from Cattle in Uruguay Reveal the Circulation of Common and Uncommon Genotypes and Suggest Interspecies Transmission
Source: Pathogens. 2020 Jul 14;9(7):570. doi: 10.3390/pathogens9070570 (PMC7400708; doi:10.3390/pathogens9070570)

NSP1

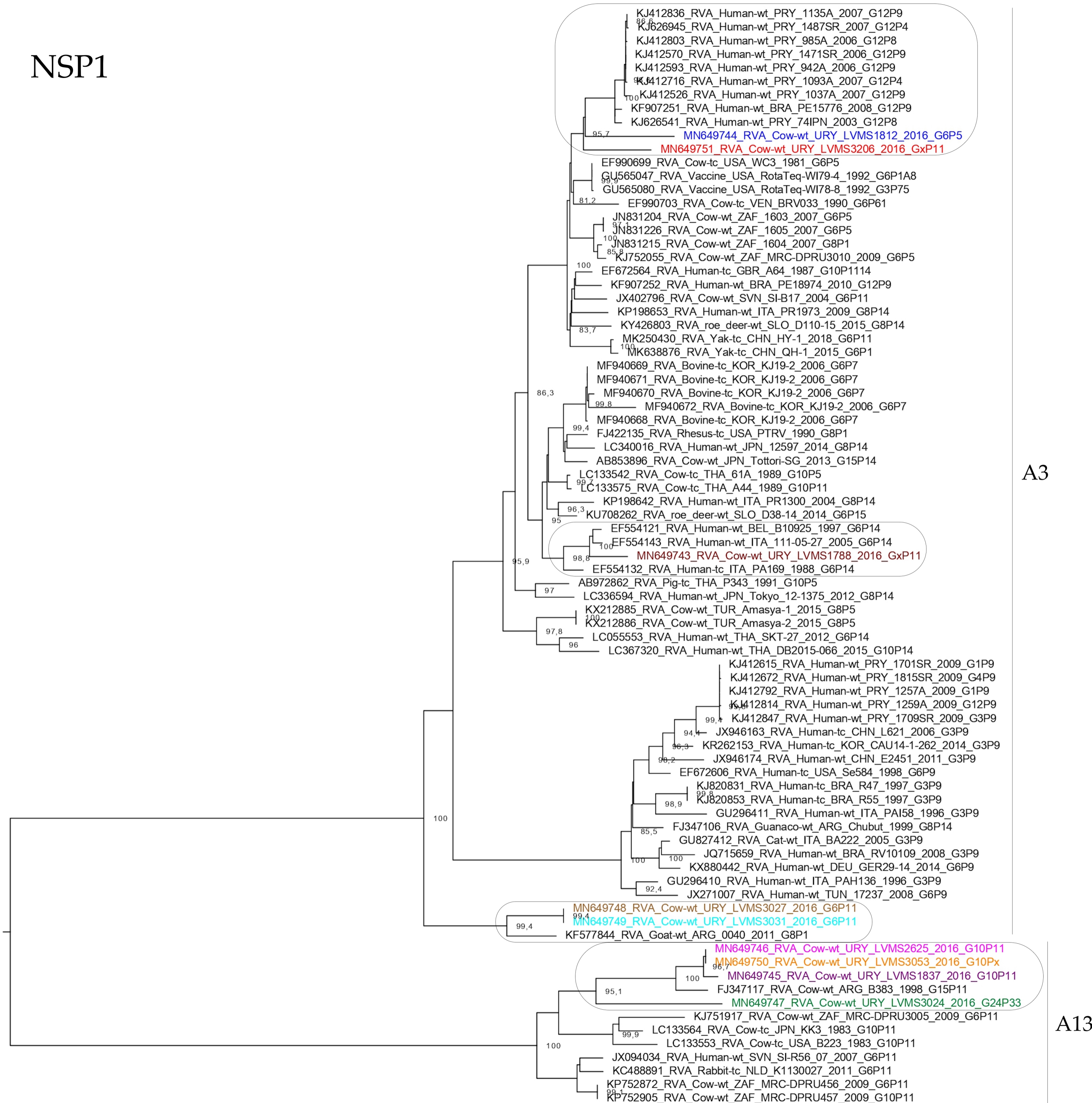

A13

Supplement: Supplementary file 1 [file pathogens-09-00570-s001.zip › Supplementary figures/Figure S1.pdf]

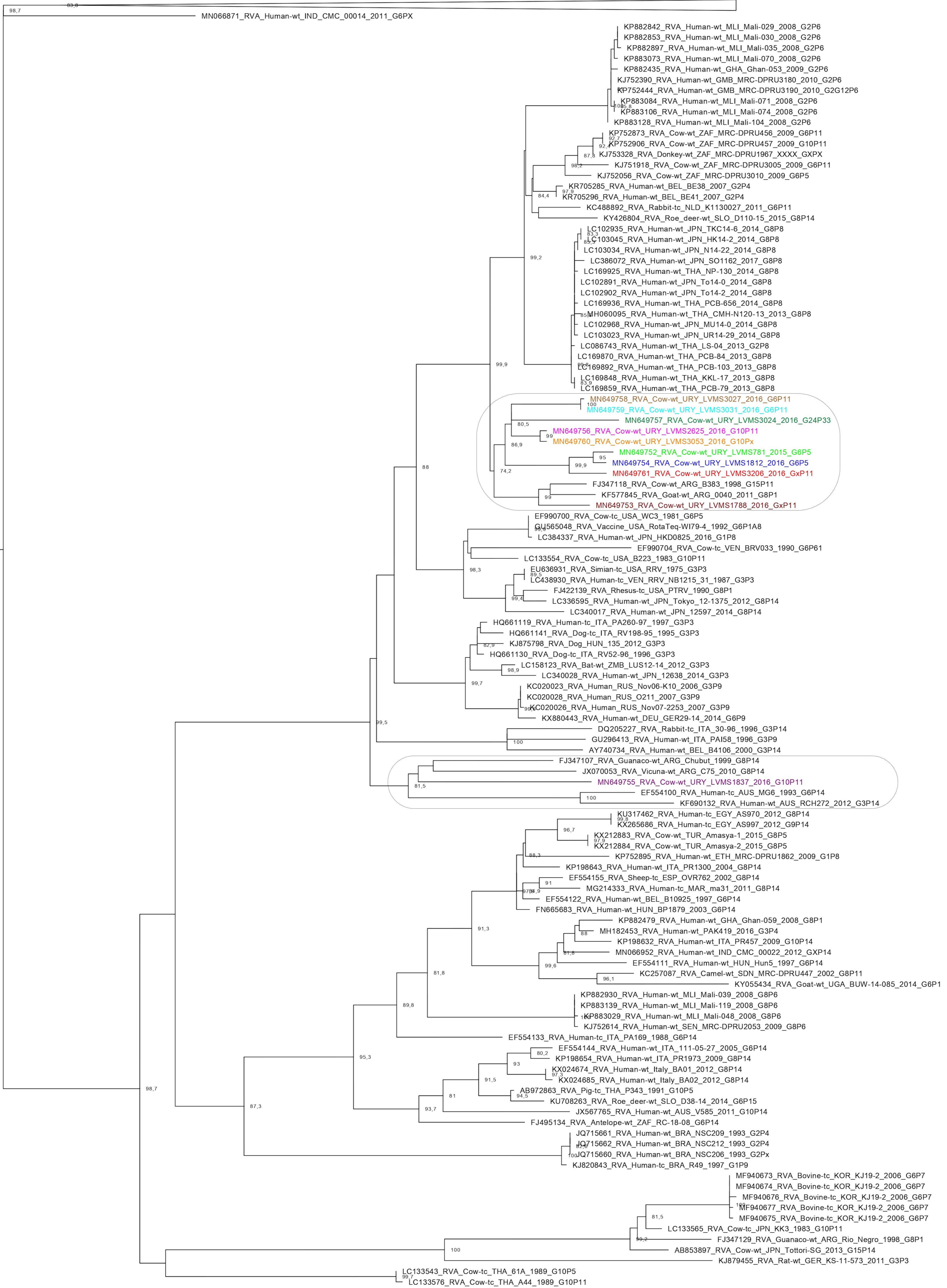

Supplement: Supplementary file 1 [file pathogens-09-00570-s001.zip › Supplementary figures/Figure S2.pdf]

NSP3

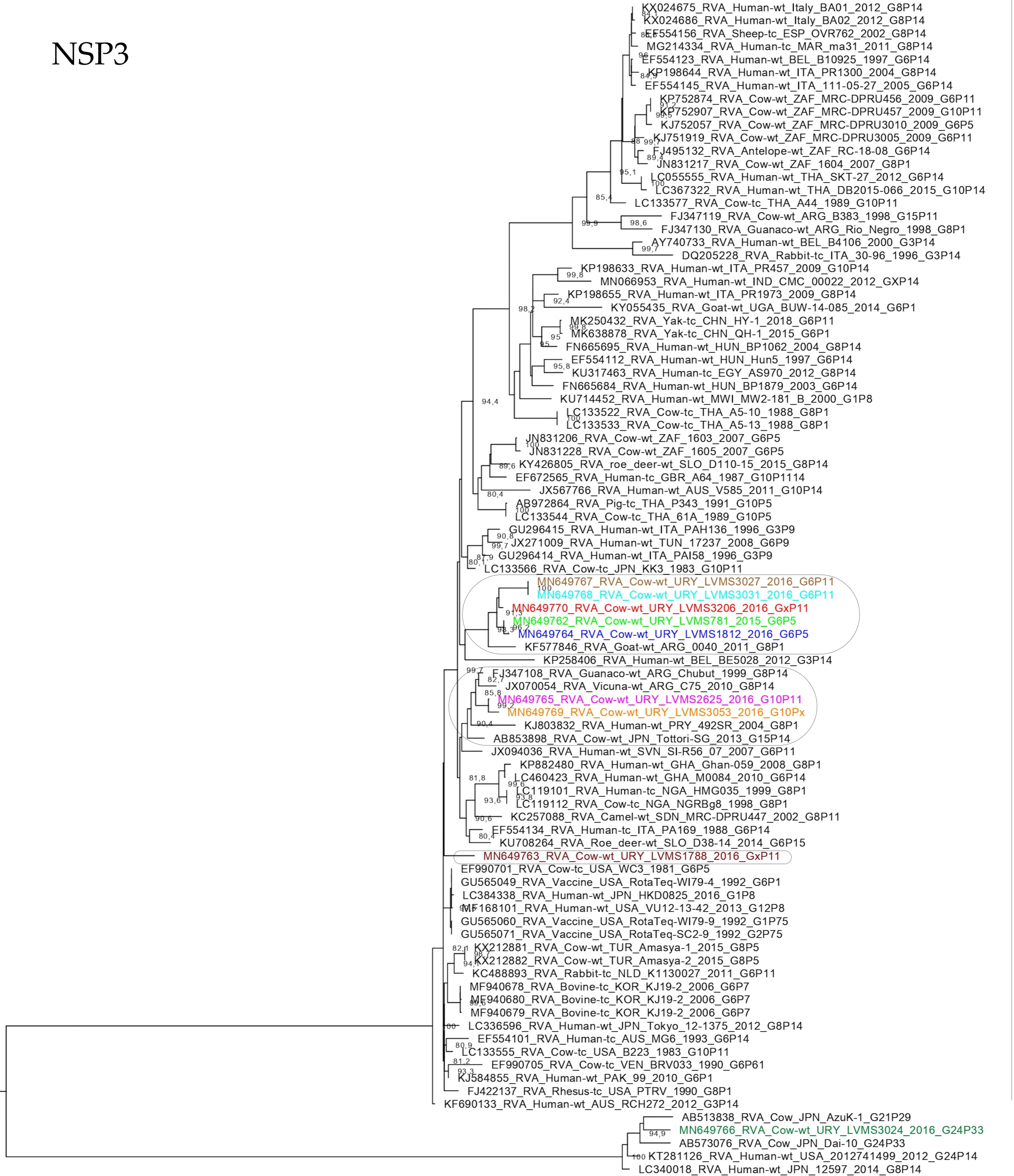

T6

T9

Supplement: Supplementary file 1 [file pathogens-09-00570-s001.zip › Supplementary figures/Figure S3.pdf]

NSP4

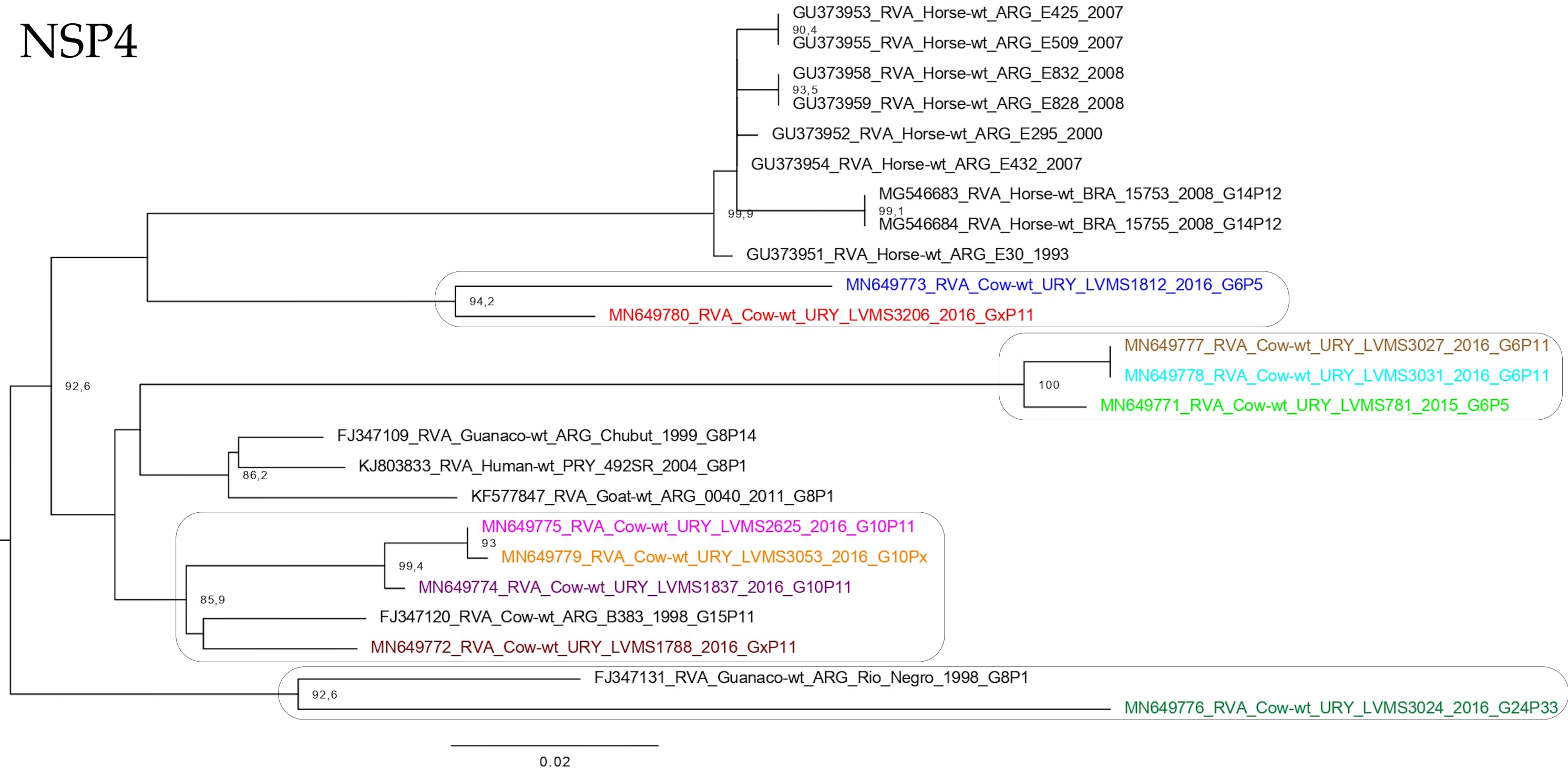

E12

Supplement: Supplementary file 1 [file pathogens-09-00570-s001.zip › Supplementary figures/Figure S4.pdf]

## NSP5

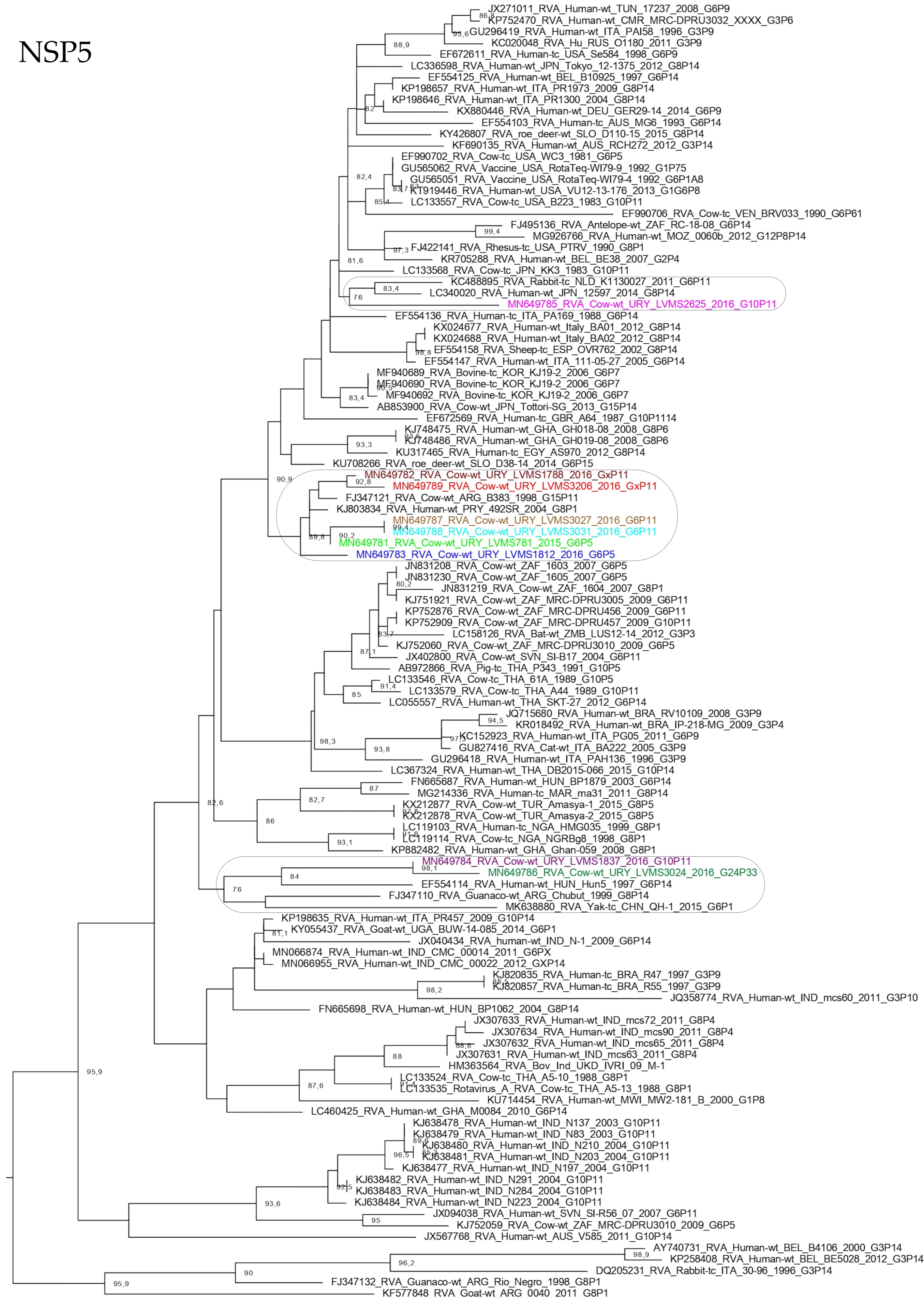

H3

0.02

Supplement: Supplementary file 1 [file pathogens-09-00570-s001.zip › Supplementary figures/Figure S5.pdf]

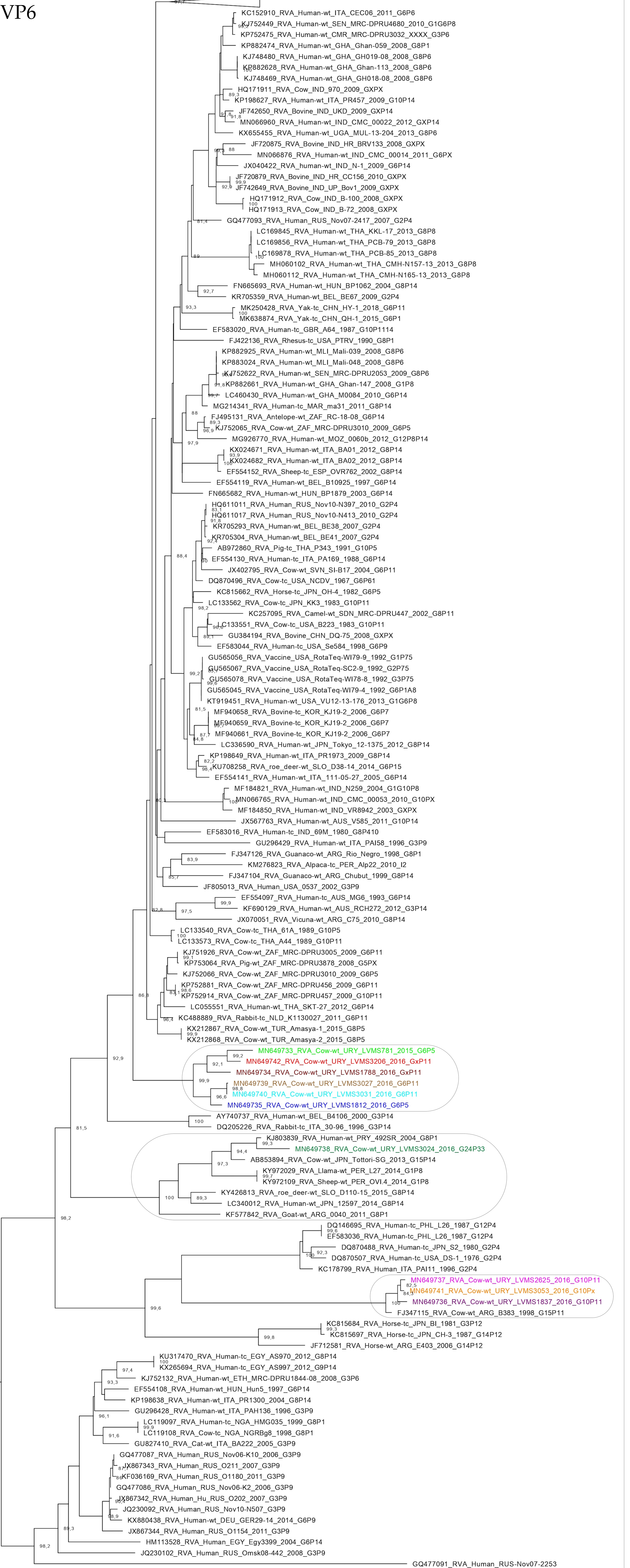

Supplement: Supplementary file 1 [file pathogens-09-00570-s001.zip › Supplementary figures/Figure S6.pdf]
